# Unravelling the Flotation Performance of 1-Hydroxy-2-naphthyl hydroxamic Acid and Styrene Phosphonic Acid Collectors on Monazite Using Experiments and DFT Calculations

**DOI:** 10.3390/molecules29051052

**Published:** 2024-02-28

**Authors:** Weiwei Wang, Zhengyao Li, Weiyao Zhu, Shaochun Hou, Chunlei Guo

**Affiliations:** 1 School of Civil and Environmental Engineering, University of Science and Technology Beijing, Beijing 100083, China; 2 State Key Laboratory of Bayan Obo Rare Earth Resource Researches and Comprehensive Utilization, Baotou Research Institute of Rare Earths, Baotou 014030, China; 3 State Key Laboratory of High-Efficient Mining and Safety of Metal Mines of Ministry of Education, University of Science and Technology Beijing, Beijing 100083, China

**Keywords:** monazite, DFT, 1-hydroxy-2-naphthyl hydroxamic acid, styrene phosphonic acid, adsorption energies, flotation

## Abstract

The atomic-level structure and electronic properties of monazite were investigated using a first-principles method based on density functional theory (DFT). First, the geometric structure of monazite was optimized, followed by calculations of its Mulliken population, electron density, and density of states, which were subsequently analyzed. The findings of this analysis suggest that monazite is highly susceptible to cleavage along the {100} plane during crushing and grinding. When SPA was utilized as the collector, the recovery rate of monazite was higher than that when LF-P8 was used. The zeta potential and adsorption energy results indicated that the zeta potential after SPA adsorption tended towards negativity, and the adsorption energy was smaller, indicating that SPA exhibited stronger adsorption performance. LF-P8 was stably adsorbed on the monazite (100) surface via mononuclear double coordination. SPA was stably adsorbed on the surface of monazite (100) via binuclear double coordination. The results of this study provide valuable insights into the adsorption of monazite by commonly used flotation collectors. These findings are of substantial importance for future endeavors in designing flotation collectors capable of achieving selective monazite flotation.

## 1. Introduction

The rare earth elements, comprising 17 elements, including 15 lanthanide elements, yttrium, and scandium, possess unique physical and chemical properties, such as magnetic, optical, and electrical characteristics, as a result of their distinctive electronic shell structures [1,2]. These elements have emerged as indispensable core basic materials and play a vital role in various emerging strategic industries, including new energy vehicles, industrial robots, electronic information, aerospace, national defense and military, energy conservation and environmental protection, and high-end equipment manufacturing [3,4,5]. Developed countries and regions, such as Europe, the United States, and Japan, have recognized the strategic importance of rare earth elements and have included them in their “strategic elements of the 21st century” lists; they have formed strategic reserves and conducted key studies accordingly [6,7].

Monazite is a phosphate rare earth mineral with the chemical formula (Ce, La, Nd, Th) PO4 [8]. It is a primary source of rare earth elements, including cerium, lanthanum, praseodymium, and neodymium [9,10]. Monazite is typically associated with minerals that contain calcium, such as fluorite, apatite, and dolomite [10,11,12]. A highly efficient flotation process is the preferred method for monazite recovery [13]. This process utilizes the difference in surface hydrophobicity between valuable minerals and gangue minerals to separate them. However, to increase the surface hydrophobicity of valuable minerals and reduce the difference between the surface hydrophobicity of valuable and gangue minerals, collectors such as fatty acids, phosphonic acids, and hydroxamic acids are necessary [14,15,16,17].

The carboxylic group of fatty acid collectors, including oleic acid, can react with metal ions present on the mineral surface, resulting in the formation of metal ion carboxylates [18]. The solubility of this metal ion carboxylate is inversely proportional to the strength of fatty acid adsorption on the mineral surface; that is, a lower solubility corresponds to stronger adsorption. Notably, the solubility of the carboxylate of Ce^3+^ ions is similar to that of Ca^2+^ ions, and monazite is commonly associated with calcium (Ca)-containing minerals, which leads to unsatisfactory selectivity in the flotation of monazite and limits the application of fatty acid collectors [12]. Phosphoric acid collectors and hydroxamic acid are two collectors commonly used in rare earth mineral flotation. Zhou et al. investigated the flotation performance of monoalkyl phosphate on bastnaesite and discovered its effectiveness under alkaline conditions [19]. Zhang et al. conducted flotation experiments on a Weishan rare earth ore using styrene phosphate as a collector, resulting in the production of a high-quality rare earth concentrate [20]. Ren et al. explored the flotation effect of hydroxamic acids with varying carbon chains on bastnaesite and achieved favorable separation efficiency and industrial applications [21]. Numerous scholars have also analyzed and tested the differences in surface properties before and after the interactions between the two types of collectors and rare earth minerals using methods such as infrared spectroscopy and X-ray photoelectron spectroscopy [22,23,24]. However, these studies have primarily focused on the macroscopic mechanism of the interaction between reagents and minerals, providing only qualitative insights.

The first-principles method based on density functional theory (DFT) has demonstrated its efficacy in determining the structural and electronic properties of minerals, as well as the molecular properties of flotation reagents [25]. This computational approach provides powerful theoretical support for the corresponding experimental findings. Zhou et al. [26] utilized DFT to explore the interaction between Cr^3+^ and the calcite surface, revealing that the chemical properties of the O atoms on the calcite surface were active. The adsorption position of Cr^3+^ on the surface had a substantial impact on the adsorption properties. Additionally, He et al. [27]. employed DFT to analyze the electronic properties of spodumene and subsequently investigated its floatability based on the calculated results. These results indicate that the Li-O bond is the weakest during the dissolution process. O atoms are highly reactive and readily bind to water molecules, resulting in a relatively high concentration of lithium on the surface of spodumene. However, the low activity and low charge of Li collectors lead to poor adsorption capacity and, consequently, poor floatability of spodumene when sodium oleate is used as a collector. Chen et al. [28] employed density functional theory to examine the effects of surface properties and lattice defects on sulfide ore flotation, elucidating the reasons behind the abnormal charge distribution in mineral crystals. Studies have revealed that changes in atomic positions on the surface of pyrite (100) primarily occur around Fe atoms with unsaturated bonds, and the Fe-S interaction on the surface is closer, with Fe exhibiting more active chemical properties. The bandgap width on the surface of pyrite (100) decreases and the conductivity of the surface layer is stronger than that of the mineral crystals. However, there is limited information available in published works on the electronic properties of monazite and their impact on surface reactions during the hydroxamic acid and phosphonic acid flotation processes.

This study initially optimized the structure of monazite by utilizing DFT calculations. Subsequently, we explored the electronic properties, encompassing aspects such as the Mulliken population, electron density, density of states, and dissociation characteristics. A micro-flotation test was then performed to determine the recovery rate of each collector. This study investigated the interaction and adsorption mechanisms between 1-hydroxy-2-naphthyl hydroxamic acid (LF-P8) and styrene phosphonic acid (SPA) on the surface of monazite (100) to calculate the adsorption energy and determine the affinity of these collectors. The chemical reactivity of LF-P8 and SPA with the monazite surface was analyzed using density of states and Mulliken charge family analysis. Finally, this study explored the concept of validating the predicted adsorption energy of DFT with the micro-flotation recovery rate to identify high-performance and selective collectors before laboratory synthesis.

## 2. Results and Discussion

### 2.1. Structural Properties

#### 2.1.1. Mulliken Population Analysis

The valence electron configurations of Ce, P, and O free atoms in monazite before optimization were Ce 4f^1^ 5d^1^ 6s^2^, P 3s^2^ 3p^3^, and O 2s^2^ 2p^4^, respectively. The Mulliken populations of each atom after optimization are presented in Table 1. The optimized valence electron configuration of Ce was Ce 4f^0.19^ 5d^0.94^ 5s^2.17^, with an electron number of 3.30 e localized on the atom and a loss of 0.70 e. The charge of the Ce atom was +0.7 e, rendering it an electron acceptor, and it lost electrons from 4f. The optimized valence electron configuration of atom P was P 3s^0.93^ 3p^1.95^, with an electron number of 2.88 e localized and a loss of 2.12 e. The charge of the P atom was +2.12 e, making it an electron acceptor, and it lost electrons from its 3s and 3p orbitals. The optimized valence electron configuration of O1 was O 2s^1.85^ 2p^5.08^, with an electron number of 6.93 e localized and a gain of 0.93 e. The charge of the O1 atom was +2.12 e, as an electron donor, and electrons were obtained in 2p. The optimized valence electron configuration of the oxygen molecule O2 was O 2s^1.85^ 2p^5.07^, with an electron number of 6.92 e and a charge of −0.92 e. This indicated that O2 acted as an electron donor and acquired electrons in 2p. Similarly, the optimized valence electron configuration of the oxygen molecule O3 was O 2s^1.85^ 2p^5.09^, with an electron number of 6.93 e and charge of −0.93 e. The O3 atom also functioned as an electron donor and gained electrons in 2p. Finally, the optimized valence electron configuration of the oxygen molecule O4 was O 2s^1.85^ 2p^5.08^, with an electron number of 6.93 e and a charge of −0.93 e. Similar to O2 and O3, O4 acted as an electron donor and obtained electrons in 2p.

The Mulliken bond population of monazite are presented in Table 2. The population of P-O1 bonds in the phosphate group was 0.66, with a bond length of 1.524 Å. Similarly, the population of the P-O2 bond was 0.66, with the same bond length. The O3 bond population was 0.65, and the bond length was 1.524 Å. The P-O4 bond had a population of 0.65 and a bond length of 1.524 Å. The high population value of the P-O bond indicated a covalent bond with high bond strength, making it difficult to rupture under an external force. The population values of Ce-O bonds were low, with Ce-O1, Ce-O2, Ce-O3, and Ce-O4 bonds having population values of 0.25, 0.24, 0.24, and 0.24, respectively. This suggested that Ce was connected to the O atom of PO_4_^3−^ through an ionic bond. The Ce-O bond was more likely to rupture during crushing and grinding.

#### 2.1.2. Electron Density and Density of States Analysis

**Figure 1:**
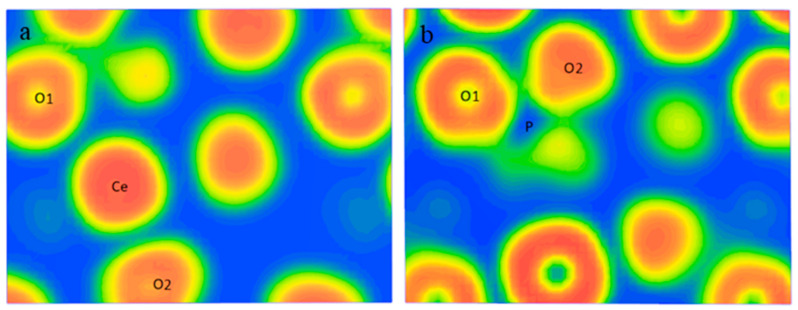
Electron density maps of Ce-O (**a**) and P-O (**b**).

Figure 1 illustrates significant electron overlaps between the P and O atoms in PO_4_^3−^, suggesting that a substantial number of shared electrons between P-O and P-O bonds were in covalent bonds. Conversely, there was minimal electron overlap between Ce and O atoms. There was almost no electron cloud overlap between the Ce and O atoms, with electrons primarily congregating near the O atoms, which possessed stronger electronegativity. This phenomenon may be attributed to the fact that Ce atoms lost electrons from their 4f orbitals and became positively charged, whereas O atoms gained electrons in their 2p orbitals and became negatively charged. This resulted in the formation of a Ce-O connection through electrostatic attraction, exhibiting ionic bonding properties. The results of the charge density analysis were aligned with those of the bond population analysis.

**Figure 2:**
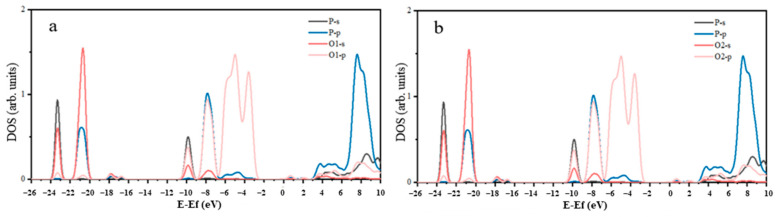
Density of states of P-O1 (**a**) and P-O2 (**b**).

**Figure 3:**
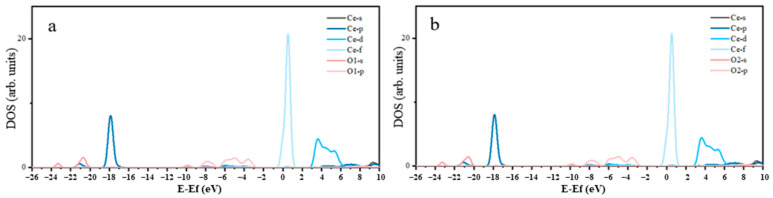
Density of states of Ce-O1 (**a**) and Ce-O2 (**b**).

Figure 2 shows the density of states of the P-O atoms. The P-O bonding was mainly attributed to the strong hybridization of O2p orbitals and P3p orbitals in the energy range of −8.82 eV to −6.72 eV. The overlap of the density of states peaks of the O2p and S3p orbitals covered a substantial area, resulting in a strong covalent P-O bond. In Figure 2, it can be observed that the 2p orbit of the O atom and the 5d orbit of the Ce atom overlapped in the ranges of −8.75 eV to −6.72 eV and −6.21 eV to −2.63 eV, respectively. However, the contribution of the Ce5d orbital to the O2p orbital was weak, causing the Ce-O bond to exhibit weak covalency, suggesting the nature of an ionic bond. According to the peak near the Fermi level in Figure 3, one peak of Ce 4f passed through the Fermi level and had considerable intensity, indicating that Ce atoms were highly active in monazite. The electronic activity of the Ce atom was significantly higher than that of the O atom, leading to greater chemical activity.

#### 2.1.3. Cleavage Characteristics of Monazite

**Figure 4:**
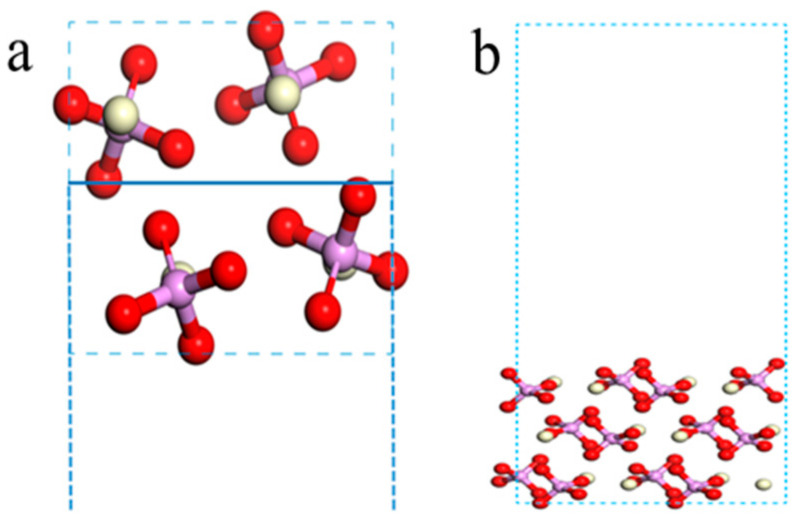
Cleavage planes along the {100} direction (**a**) and the structure of the {100} cleavage surface (**b**).

As shown in Table 3, the order of unsaturated bond density in the monazite crystal faces was {100} > {101} > {210} > {001}. Notably, the unsaturated bond density of the {100} plane was the lowest among the four crystal faces examined at only 2.20 nm^−1^. The layer spacing was greater than that of the {111} and {110} planes and was nearly identical to that of the {120} plane. Consequently, monazite was most susceptible to cleavage along the {100} plane under the effect of external forces. The unsaturated bond density of the {120} plane surpassed that of the {100} plane and possessed the largest layer spacing. Therefore, the crystal was prone to fracture along this plane when subjected to an external force. Figure 4a,b depict the orientations of the {100} and {120} planes in the monazite cells, respectively. As can be inferred from the figure, Ce^3+^ and PO_4_^3−^ were distributed in the same layer in a 1:1 ratio on these two crystal faces. The {100} and {120} planes were electrically neutral, which facilitated the cleavage. The {111} and {110} planes were less likely to undergo cleavage due to the high number of unsaturated bonds on the crystal faces. As illustrated in Figure 3, on the {100} and {120} cleavage planes, the ratio of Ce^3+^ to PO_4_^3−^ was 1:1, and the atomic number ratio of Ce and O exposed to the surface was 1:2. Given that the surface was dominated by electronegative O, monazite exhibited strong electronegativity.

### 2.2. Micro-Flotation Experiments

**Figure 5:**
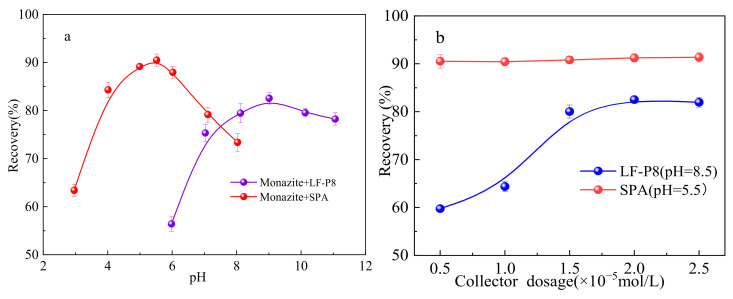
Monazite recovery as a function of pH (**a**) and dosages (**b**) of LF-P8 and SPA.

Figure 5a illustrates the effect of pH on the recovery of monazite using 2.0 × 10^−5^ mol/L LF-P8, 2.0 × 10^−5^ mol/L SPA, and 20 mg/L terpineol. In the examined pH range, the recovery of monazite using LF-P8 as a collector exhibited a statistically significant variation when compared to that using SPA. When SPA was used as the collector, the recovery of monazite increased with increasing pulp pH from 3 to 5.5, reaching a maximum value of approximately 90% at pH 5.5, after which it declined as the pH continued to rise. In contrast, when LF-P8 was used as the collector, the recovery of monazite increased to approximately 82% at pH 6–9 and then rapidly decreased as the pH continued to rise. These results indicated that alkaline enhancement was detrimental to the flotation of monazite with SPA as the collector, whereas LF-P8 exhibited the most effective flotation performance in weakly alkaline systems.

Figure 5b depicts the results of the experiments conducted to determine the recovery of monazite in the presence of various dosages of LF-P8 and SPA with a terpineol concentration of 20 mg/L. Within the range of SPA collector dosages investigated, the recovery of monazite remained constant at approximately 90%. When SPA was utilized as the collector, the recovery of monazite increased significantly with increasing dosage and reached a maximum value of 82.55% at a dosage of 2.0 × 10^−5^ mol/L. Additionally, the recovery of monazite tended to remain stable as the collector dosage was further increased. Notably, the recovery of monazite achieved using the SPA collector was higher than that obtained with LF-P8 in the range of collector dosages studied.

### 2.3. Zeta Potential Measurements

**Figure 6:**
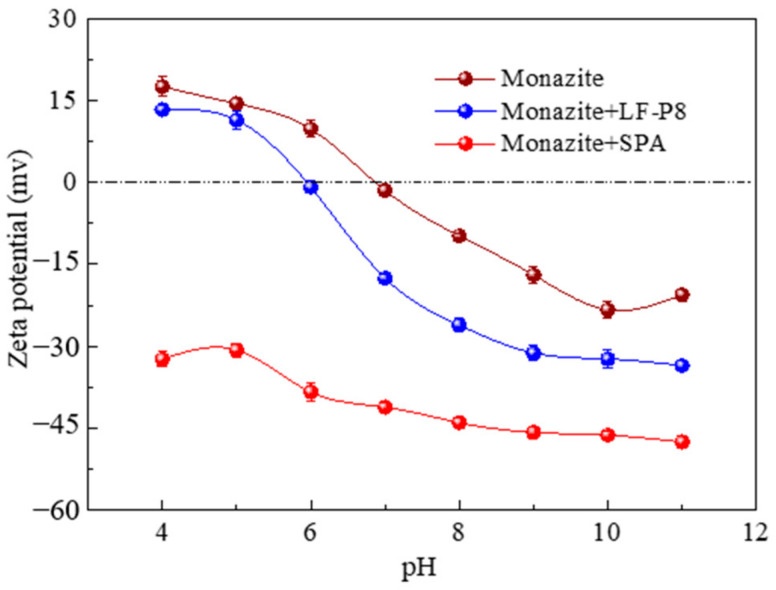
Zeta potentials of monazite as a function of pH in the absence and presence of dosages of LF-P8 and SPA.

The zeta potential is a crucial parameter in flotation for characterizing the surface properties of mineral particles because it depends on the surface charge of mineral particles and the adsorbed flotation reagents on the particle surface [29]. The zeta potentials of the monazite samples in the absence and presence of LF-P8 and SPA are shown in Figure 6. The isoelectric point (IEP) of the bare monazite was 6.8, which was consistent with previous studies [30,31]. At pH values greater than 6.8, the monazite surface was negatively charged. When LF-P8 was added, the zeta potential of the monazite samples decreased to 2.2, and the zeta potential of rutile decreased overall, especially in the pH range of 7–10, indicating that LF-P8 anions were adsorbed onto the rutile surface. In the presence of SPA, the zeta potential of the monazite samples tended to be more negative than that in the presence of LF-P8, indicating that the adsorption of SPA onto the surface of monazite was stronger than that of LF-P8, which was consistent with the results of the flotation experiment.

### 2.4. Adsorption Mechanism of LF-P8 and SPA on the Monazite (100) Surface

#### 2.4.1. Geometries and Adsorption Energies of LF-P8 and SPA Collectors on the Monazite (100) Surface

**Figure 7:**
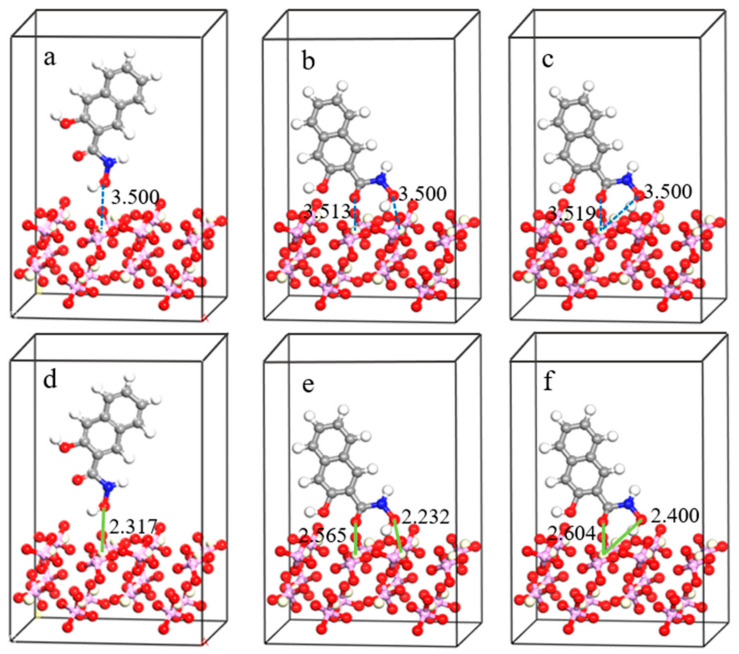
Different adsorption forms of LF-P8 on the monazite (100) surface: (**a**,**d**) mononuclear single coordination; (**b**,**e**) binuclear double coordination; (**c**,**f**) mononuclear double coordination.

**Figure 8:**
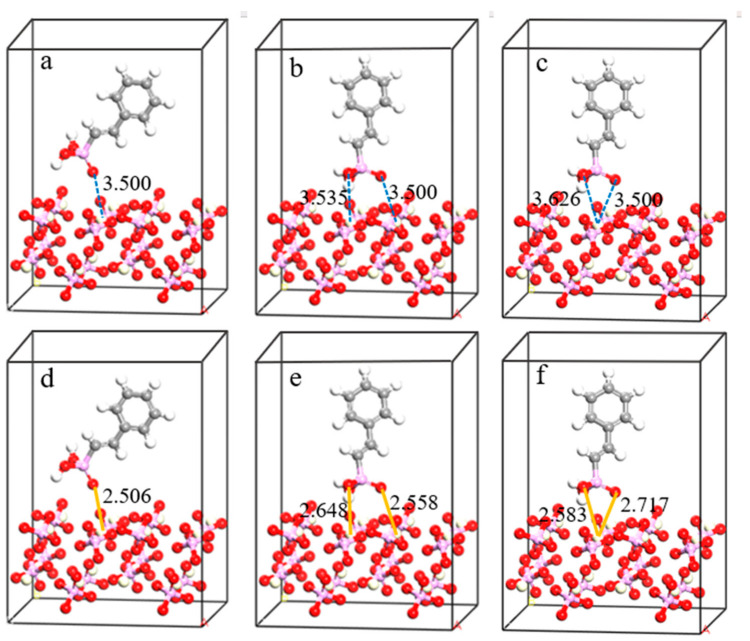
Different adsorption forms of SPA on the monazite (100) surface: (**a**,**d**) mononuclear single coordination; (**b**,**e**) binuclear double coordination; (**c**,**f**) mononuclear double coordination.

To investigate the adsorption mechanism of LF-P8 and SPA on the monazite (100) surface, these two reagents were arranged in various adsorption forms on the monazite (100) surface for optimization calculations. The configurations of adsorption, both prior to and following adsorption on the monazite (100) surface, are depicted in Figure 7 and Figure 8. The adsorption energy varies when different molecules or ions interact with the same mineral surface and can be utilized to ascertain the strength of the bond between the agent and mineral surface. The formula for calculating the adsorption energy of the reagents on mineral surfaces is as follows:

ΔE = E_com_ − E_surf_ − E_rea_ (1)

where ΔE represents the adsorption energy and E_com_, E_surf,_ and E_rea_ represent the total energy of the optimized adsorption complex, surface structure, and adsorbate, respectively. When the adsorption energy is negative, adsorption can occur spontaneously [32]. The smaller the adsorption energy is, the more stable the adsorption is. Conversely, when the adsorption energy is zero or positive, adsorption cannot occur spontaneously. The calculated adsorption energies of LF-P8 and SPA with different adsorption forms on the surface of monazite (100) are listed in Table 4. Among the various models, the adsorption energy of LF-P8 was the lowest in the single-nucleus double-coordination model. Hence, the final stable adsorption form of LF-P8 on the monazite (100) surface was the formation of a five-membered-ring single-nucleus double-coordination configuration. The adsorption energy of SPA was the smallest in the binuclear double coordination model, suggesting that SPA formed hydrogen and O_SPA_-Ce bonds after adsorption on the monazite (100) surface.

#### 2.4.2. Electronic Structures of LF-P8 Collector on the Monazite (100) Surface

The mechanism of LF-P8 adsorption on the (100) surface of monazite was examined by analyzing the changes in the Mulliken charge population and density of states before and after the reaction between the O atom on the oxime group of LF-P8 and the Ce atom on the surface of monazite. The Mulliken charge population values before and after adsorption of the O and Ce atoms are listed in Table 5. As shown in Table 5, upon adsorption of LF-P8 on the surface of monazite (100), the 2s orbitals of the O1 atom, 2s and 2p orbitals of the O2 atom, 5p orbitals of the Ce1 atom, and 6s, 5p, 5d, and 4f orbitals of the Ce2 atom all lost electrons, whereas the 2p orbitals of the O1 atom and 5d and 4f orbitals of the Ce1 atom gained electrons. This indicated a reduction in the electron count of the O2 atom, a decrease in the electron count of the Ce2 atom, and an increase in the electron count of the Ce1 atom. Consequently, the negative charge of the O atom decreased, while the positive charge of the Ce atom increased. The phenomenon of electron transfer may be attributed to the proximity of the O nucleus to the Ce nucleus during the bonding process, which contributed to the interaction between both nuclei to excite and transition some of the inner s and f orbitals of both atoms to p orbitals. Overall, the O atoms exhibited electron gain, whereas the Ce atoms experienced electron loss.

**Figure 9:**
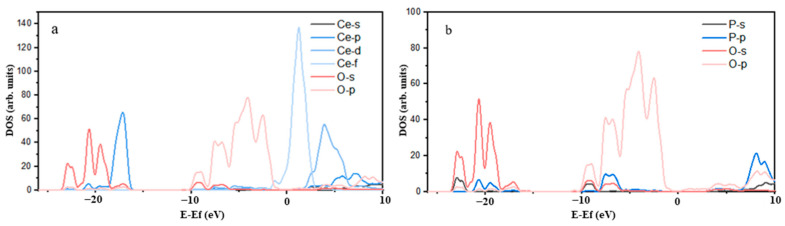
Density of states of Ce-O (**a**) and P-O (**b**) after adsorption LF-P8.

The density of states for P-O and Ce-O after adsorption is shown in Figure 9. Evidently, the 2p orbitals of the adsorbed O atom significantly contributed to the peaks near the Fermi level. Some of these peaks intersected with the Fermi level and entered the conduction band region, demonstrating the strong catalytic activity of the adsorbed O atom, which was primarily provided by the 2p orbitals. The 4f orbitals of the adsorbed Ce atom primarily contributed to the peaks in the density of states near the Fermi level, with certain 5p, 5d, and 4f orbitals crossing the Fermi level. This suggested that the catalytic activity of the adsorbed Ce atom was provided by the 5p, 5d, and 4f orbitals. Based on the aforementioned Mulliken charge population and density of states analyses, after adsorption, the stability of the O and Ce atoms in LF-P8 increased, and a chemical bond in the form of an O-Ce coordination bond was established between them. The formation of this bond was primarily attributed to the participation of the 2p orbital electrons of the O atom and the 6s and 5d orbital electrons of the Ce atom in the reaction.

#### 2.4.3. Electronic Structures of SPA Collector on the Monazite (100) Surface

An analysis of the Mulliken charge population and changes in the density of states before and after the reaction between the double-bond O atom of SPA and the Ce atom on the surface of monazite elucidated the adsorption mechanism of SPA on the (100) surface of monazite. The Mulliken charge population values for O and Ce atoms, both pre- and post-adsorption, are listed in Table 6. As indicated in Table 6, following the adsorption of SPA on the monazite (100) surface, there was a decrease in the electrons of O1 and O2 atoms, whereas an increase was observed in the electrons of Ce1 and Ce2 atoms. This resulted in a reduction in the negative charge value of O atoms and an increase in the positive charge value of Ce atoms. The observed change can be attributed to the proximity of the O1 nucleus to the Ce nucleus during the bonding process. The interaction between both nuclei prompted certain inner s orbitals and f orbitals of both atoms to be excited and transition to p orbitals, leading to an overall gain of electrons in the O atom and a loss of electrons in the Ce atom.

**Figure 10:**
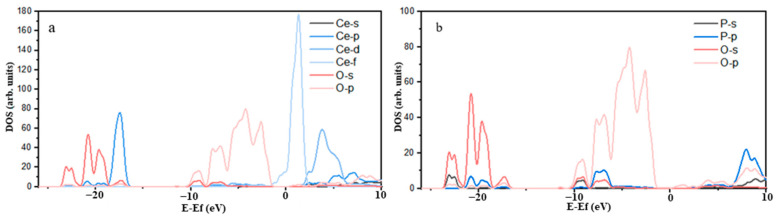
Density of states of Ce-O (**a**) and P-O (**b**) after adsorption SPA.

Changes in the atomic density of states after adsorption are shown in Figure 10. The 2p orbitals of the adsorbed O atom primarily contributed to the peaks in the density of states near the Fermi level. Furthermore, some of the peaks in the density of states of the 2p orbitals extended beyond the Fermi level and entered the conduction band region, suggesting that the O atom exhibited substantial activity prior to adsorption, which was primarily attributed to the 2p orbitals. The primary contribution to the peaks in the density of states near the Fermi level arose from the 4f orbitals of the adsorbed Ce atom. Furthermore, certain 5p, 5d, and 4f orbitals crossed the Fermi level, indicating that the reaction activity of the adsorbed Ce atoms was affected by the 5p, 5d, and 4f orbitals. The above Mulliken charge population and density of states analyses revealed that the adsorption process results in a more stable state for both the O and Ce atoms, with the formation of an O-Ce coordination bond between them. The formation of this chemical bond was mainly attributed to the participation of 2p orbital electrons from the O atom and 5p and 5d orbital electrons from the Ce atom in the reaction.

## 3. Materials and Methods

### 3.1. Materials

**Figure 11:**
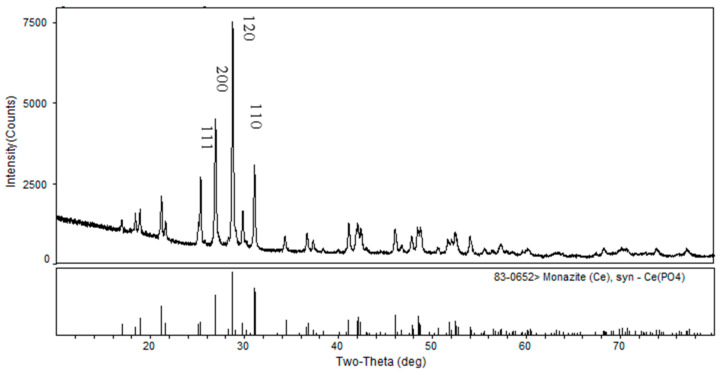
XRD spectrum of monazite.

Monazite samples were obtained from Weishanhu (Shandong, China). Initially, the artificially crushed samples were finely ground using a ceramic ball mill, followed by wet sieving to obtain a particle size of −74 + 37 μm. Subsequently, pre-enrichment was performed using a shaking table, and separation was performed using a magnetic separator to obtain pure monazite minerals. Figure 11 illustrates the X-ray diffraction (XRD) spectrum of monazite, which confirms its high purity. The diffraction peaks corresponding to the (111), (200), (120), and (110) planes were more easily mechanically crushed and ground. The fraction with a size of −75 + 37 μm was utilized for the micro-flotation experiments, whereas the fraction with a size below 5 μm was employed for zeta potential measurements.

**Figure 12:**
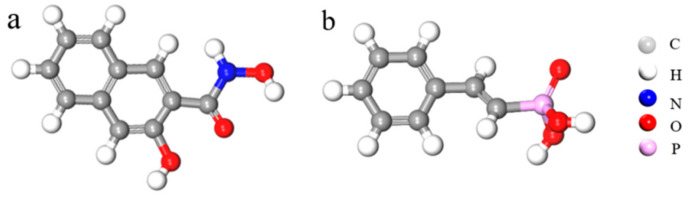
Collector structures of 1-hydroxy-2-naphthyl hydroxamic acid (**a**) and styrene phosphonic acid (**b**).

The styrene phosphonic acid (Figure 12b), pH adjuster HCl, and NaOH used in the experiment were procured from Aladdin Reagent Co., Ltd. (Shanghai, China), and their analytical purity was assessed. The 1-hydroxy-2-naphthyl hydroxamic acid (Figure 12a) and terpineol were sourced from Baoshan Mining Co., Ltd., Baogang Group, Baotou, China. All experiments were performed using deionized water with a conductivity of 2.0 × 10^−5^ S/m.

### 3.2. Micro-Flotation Experiments

The equipment used for the flotation process was an XFG single-cell flotation machine manufactured by Wuhan Rock Machinery Factory, Wuhan, China. The flotation cell capacity was 40 mL. In each test, a 2.0 g monazite ore sample was placed in 30 mL of deionized water. After stirring for 3 min, the pH regulator, collector LF-P8 or SPA, and foaming agent terpineol were sequentially added. After the addition of each agent, the mixture was stirred for 3 min. Finally, the foam product and the product remaining in the cell were separately dried and weighed for 3 min for flotation scraping. The recovery rate was calculated. Each flotation experiment was repeated thrice, and the average and standard deviation were recorded [33].

### 3.3. Zeta Potential Measurements

The zeta potential of the monazite was determined using an automatic potential analyzer equipped with a sample cell. A purified mineral sample (2.0 g) was added to 30 mL of 1 for each measurement. KCl (10^−3^ mol/L) was used as the background electrolyte. The pH was adjusted using sodium hydroxide or sulfuric acid, followed by the addition of LF-P8 or SPA according to the testing requirements. The mixture was stirred for 3 min and allowed to stand for more than 30 min, and the supernatant was collected for subsequent ζ potential measurements.

### 3.4. Calculation Methods

In this study, all calculations were performed using the CASTEP code [34], which employs DFT and the plane wave pseudopotential method. The exchange correlation potential was described using the PBE functional within the Generalized Gradient Approximation (GGA). The cutoff value for the plane wave energy was set at 450 eV, and the tolerance for self-consistent calculations was set at 5.0 × 10^−5^ eV/atom. Geometric optimization was conducted with maximum displacement thresholds of less than 0.005 Å and maximum atomic force thresholds of less than 0.1 eV/Å.

**Figure 13:**
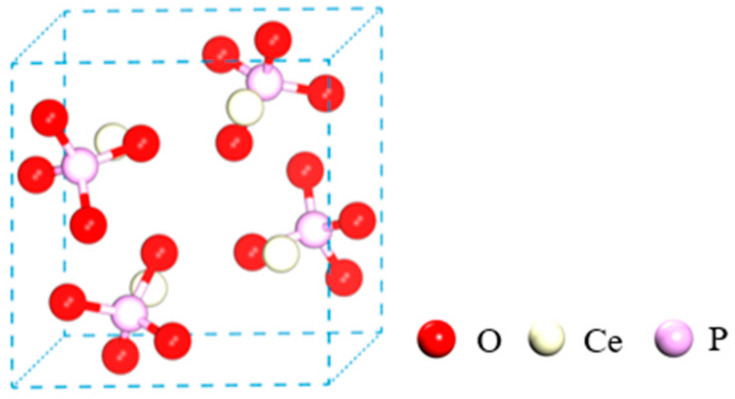
Crystal structure of the monazite bulk model.

The initial crystal structure of monazite was proposed by Ueda in 1967 and Ghouse in 1968 [35,36]. They accurately predicted the atomic arrangement based on crystal chemical parameters. Subsequently, the parameters of the monazite structure have been continuously reported and refined. The calculation model was established using the lattice parameters provided by NI in the American Mineralogist Crystal Structure Database (AMCSD), which is a widely referenced source [37]. Monazite is an island phosphate mineral in the monoclinic crystal system. In the crystal structure, the [PO_4_] tetrahedrons are arranged in isolated islands and connected by Ce^3+^ ions at the apex of the tetrahedron. Each Ce^3+^ ion is surrounded by seven [PO_4_] tetrahedrons and connected to nine O atoms at the apex of the tetrahedron, with a coordination number of 9. The PO_4_^3−^ complex anion, with a radius of 0.153 nm, forms a stable structure with Ce^3+^, which has a larger radius. Monazite crystals belong to the monoclinic crystal system and possess a P21/C (C2H-5) space group. Monazite’s cell parameters, as shown in Table 7, are a = 6.790 A, b = 7.020 A, and c = 6.467 A, with α = γ = 90° and β = 103.38°. The crystal structure of the monazite was optimized, as shown in Figure 13. The optimized lattice parameters are listed in Table 7. As can be observed from the table, the theoretically calculated values of the monazite lattice constants a, b, and c aligned closely with the experimental values, with deviations within the 1% range. The calculation results were consistent with those of the experiments, which proved the reliability of the abovementioned calculation method and process.

The construction and optimization of the (111), (100), (120), and (110) surfaces were accomplished using the BuildSurface module in the MS. To determine the most stable surface model, convergence tests were conducted for the atomic layer number and the vacuum layer thickness. Subsequently, Equations (2) and (3) were applied to determine the fracture bond density and surface energy of various crystal faces of monazite [38,39].

(2) $$D_{b}=\frac{N_{b}}{s}$$

(3) $$E_{surface}=\frac{E_{slab}-\left(\frac{N_{slab}}{N_{bluk}}\right)E_{bluk}}{2A}$$

Here, *D_b_* and *N_b_* represent the crystal plane fracture bond density and the unit plane fracture bond number, respectively. *S* represents the area of the crystal face, and *E_slab_* and *E_bulk_* denote the total energies of the surface structure and bulk phase cells, respectively. Nslab and *N_bulk_* refer to the surface structure and the total number of atoms per cell, respectively. Finally, *A* represents the area of the surface structure along the Z axis.

## 4. Conclusions

Monazite P-O bonds are covalent, which makes them difficult to break under external forces. During crushing and grinding, the Ce-O bond population was more susceptible to breakage. The order of unsaturated bond density on each crystal plane of the monazite was {100} > {101} > {210} > {001}. On the {100} cleavage surface, the ratio of Ce^3+^ to PO4^3−^ was 1:1, and the atomic ratio of Ce to O exposed on the surface was 1:2, with negatively charged O being the main component. As a result, the negative charge of the monazite was strong.

The results of both types of collector microbubble flotation experiments demonstrated that the recovery of monazite can reach approximately 90% at an SPA dosage of 0.5 × 10^−5^ mol/L, whereas the recovery was only 82.55% at an LF-P8 dosage of 2.0 × 10^−5^ mol/L. The zeta potential and adsorption energy suggested that SPA possesses a superior adsorption capacity, as compared to LF-P8, on the surface of monazite. LF-P8 was adsorbed onto the surface of monazite (100) via mononuclear double coordination. After adsorption, the O and Ce atoms in LF-P8 attained greater stability, leading to the formation of an O-Ce coordination bond. The generation of this chemical bond was primarily facilitated by the 2p orbital electrons of the O atom and the 6s and 5d orbital electrons of the Ce atom. Similarly, SPA was adsorbed on the surface of monazite (100) via binuclear double coordination. Following adsorption, the O and Ce atoms in the SPA became more stable, resulting in the formation of an O-Ce coordination bond. The formation of this chemical bond predominantly involved the 2p orbital electrons of the O atom and the 5p and 5d orbital electrons of the Ce atom.

## Figures and Tables

**Table 1:** Mulliken atomic population of monazite.

Species	s	p	d	f	Total/e	Charge/e
Ce	2.17		0.94	0.19	3.30	0.70
P	0.93	1.95	0	0	2.88	2.12
O1	1.85	5.08	0	0	6.93	−0.93
O2	1.85	5.07	0	0	6.92	−0.92
O3	1.85	5.09	0	0	6.93	−0.93
O4	1.85	5.08	0	0	6.93	−0.93

**Table 2:** Mulliken bond population of monazite.

Bonds	Population	Bond Length/Å
Ce-O1	0.25	2.454
Ce-O2	0.24	2.456
Ce-O3	0.24	2.459
Ce-O4	0.24	2.460
P-O1	0.66	1.524
P-O2	0.66	1.524
P-O3	0.65	1.524
P-O4	0.65	1.524

**Table 3:** Broken bond density of monazite crystals on various surfaces.

Surface	Formula of Unit Area, *A*	*N_b_*	*D_b_* (nm^−1^)	*D* (nm)
{111}	=0.977 × 0.955 × sin 65.19°	7	8.27	0.1968
{100}	=0.702 × 0.647 × sin 90°	1	2.20	0.2886
{120}	=0.647 × 1.529 × sin 78.14°	6	6.20	0.2904
{110}	=0.647 × 0.977 × sin 80.74°	5	8.02	0.2102

**Table 4:** Adsorption energy of LF-P8 and SPA on the monazite (100) surface.

Collector	Adsorption Form	E_surf_/eV	E_rea_/eV	E_com_/eV	E_ads_/eV
LF-P8	Mononuclear single	−47,953.81	−3427.59	−51,381.48	−0.08
	Binuclear double coordination	−47,953.81	−3427.59	−51,381.54	−0.14
	Mononuclear double coordination	−47,953.81	−3427.59	−51,381.69	−0.30
SPA	Mononuclear single	−47,953.81	−2875.50	−50,829.74	−0.43
	Binuclear double coordination	−47,953.81	−2875.50	−50,829.95	−0.64
	Mononuclear double coordination	−47,953.81	−2875.50	−50,829.84	−0.53

**Table 5:** Mulliken charge population values of O and Ce atoms before and after adsorption LF-P8.

Atom	Adsorption State	s	p	d	f	Charge
O1	Before	1.83	4.76	0.00	0.00	−0.59
O1	After	1.80	4.78	0.00	0.00	−0.59
O2	Before	1.82	4.84	0.00	0.00	−0.66
O2	After	1.80	4.70	0.00	0.00	−0.50
Ce1	Before	2.15	6.00	0.90	0.19	2.76
Ce1	After	2.15	5.86	1.08	0.34	2.58
Ce2	Before	2.16	6.01	0.97	0.42	2.44
Ce2	After	2.14	5.91	0.96	0.24	2.75

**Table 6:** Mulliken charge population values of O and Ce atoms before and after adsorption SPA.

Atom	Adsorption State	s	p	d	f	Charge
O1	Before	1.86	5.15	0.00	0.00	−1.00
O1	After	1.82	5.10	0.00	0.00	−0.93
O2	Before	1.86	5.16	0.00	0.00	−1.01
O2	After	1.87	5.08	0.00	0.00	−0.96
Ce1	Before	2.15	6.00	0.90	0.19	2.76
Ce1	After	2.17	5.95	1.01	2.21	0.67
Ce2	Before	2.16	6.01	0.97	0.42	2.44
Ce2	After	2.16	5.98	0.92	2.06	0.88

**Table 7:** Monazite lattice parameters.

Lattice Parameter	Theoretical Value (Å)	Experimental Value (Å)	Reference [33]
a	6.781	6.784	6.7902
b	6.995	6.989	7.0203
c	6.462	6.459	6.2674

## Data Availability

All data generated or analyzed during this study are included in this published article.
